# Patient and Provider Satisfaction With a Geomapping Tool for Finding Community Family Physicians in Ontario, Canada: Cross-Sectional Online Survey Study

**DOI:** 10.2196/56716

**Published:** 2024-07-09

**Authors:** Christopher Belanger, Cayden Peixoto, Sara Francoeur, Lise M Bjerre

**Affiliations:** 1 Telfer School of Management University of Ottawa Ottawa, ON Canada; 2 Institut du Savoir Montfort Ottawa, ON Canada; 3 Department of Family Medicine University of Ottawa Ottawa, ON Canada

**Keywords:** primary care, language-concordant care, web-based maps, maps, physicians, experience, language, access, accessibility

## Abstract

**Background:**

Language-concordant health care, or health care in a patient’s language of choice, is an important element of health accessibility that improves patient safety and comfort and facilitates an increased quality of care. However, prior research has found that linguistic minorities often face higher travel burdens to access language-concordant care compared to the general population.

**Objective:**

This study intended to assess patient experiences and satisfaction with an online interactive physician map that allows patients to find family physicians who speak their preferred language in and around Ottawa, Ontario, Canada, as a means of identifying areas of improvement.

**Methods:**

This study used an online survey with questions related to user satisfaction. Responses to Likert-scale questions were compiled as summary statistics and short-answer responses underwent thematic analysis. The study setting was Ottawa and Renfrew County, Ontario, and the surrounding region, including the province of Quebec.

**Results:**

A total of 93 respondents completed the survey and self-identified as living in Ontario or Quebec. Overall, 57 (61%) respondents were “very satisfied” or “somewhat satisfied” with the map, 16 (17%) were “neither satisfied nor dissatisfied,” and 20 (22%) were “very dissatisfied” or “somewhat dissatisfied.” We found no significant differences in satisfaction by preferred language, age group, physician attachment, or intended beneficiary. A total of 56 respondents provided short-answer responses to an open-ended question about map improvements. The most common specific suggestion was to show which physicians are accepting new patients (n=20). Other suggestions included data refreshes (n=6), user interface adjustments (n=23), and additional languages (n=2). Some participants also provided positive feedback (n=5) or expressed concern with their inability to find a family physician (n=5). Several comments included multiple suggestions.

**Conclusions:**

While most patients were satisfied with the online map, a significant minority expressed dissatisfaction that the map did not show which family physicians were accepting new patients. This suggests that there may be public interest in an accessible database of which family physicians in Ontario are currently accepting new patients.

## Introduction

Language-concordant health care, or health care in a patient’s language of choice, is an important element of health accessibility that improves patient safety and comfort and facilitates an increased quality of care [1,2]. Conversely, language-discordant health care can lead to worse health outcomes, including an increased risk of mortality [2,3].

This study focused on the region of Ottawa, Ontario, Canada, where 12.5% of the population reports French as their mother tongue [4]. Previous research has found that French-only speakers in some parts of Ottawa face higher travel burdens to access language-concordant care compared to English speakers [5]. To help address this discrepancy, we developed a web-based mapping tool intended to help patients find language-concordant primary care in Ottawa, Ontario. The tool, named “DocMapper” in English [6] and “TrouvezUnMedecin” in French [7], presents interactive maps of family physician practice locations in the city of Ottawa and in neighboring Renfrew County, Ontario, searchable by languages spoken (52 languages for Ottawa, 22 for Renfrew County). However, the overall use and effectiveness of this language-concordant primary care tool have yet to be evaluated.

We here present the results of a user-experience survey of the online language-concordant primary care mapping tool. Following the Quadruple Aim framework [8], our objectives were to understand the patient experience with this tool and to assess whether patients perceive it as helping to strengthen access to care. Our research questions were: (1) How satisfied are patients with the mapping tool? (2) What are their suggestions for improvement? and (3) How does patient satisfaction vary between linguistic and demographic groups?

## Methods

### Setting and Population Base

This study used a cross-sectional web-based survey to evaluate patient experiences with the online mapping tool DocMapper (in English) and TrouvezUnMedecin (in French) between November 9, 2022, and June 19, 2023. During the study period, all visitors to the website were presented with a welcome message inviting them to complete the survey, as well as additional links to the survey from within the tool (Multimedia Appendix 1). The tool contained two maps: one for family physicians in Ottawa (population: 1,017,449 [4]) and one for family physicians in Renfrew County (population: 107,522 [9]), and these residents were the tool’s intended users.

### Inclusion and Exclusion Criteria

Our survey was available on the public internet, publicized through institutional social media accounts, and shared through personal and professional research networks, meaning that it was presented to a potentially large international audience. Given that the target audience of the tool comprises Ontario and Quebec residents living near Ottawa and Renfrew County, we excluded any respondents who did not report living in Ontario or Quebec. Self-reported forward sortation areas (first 3 digits of the postal code) were used to filter which respondents were included in the study population.

### Data Collection

The study survey (Multimedia Appendix 2) consisted of 2 substantive questions: one to rate users’ overall appreciation of the map using a standard 5-point Likert scale from 1 (very dissatisfied) to 5 (very satisfied) and one free-text question inviting suggestions for improvements to the map. These were followed by demographic questions, including age group, language preference, postal code (first 3 digits), medication use, enrollment status and visit frequency with a family physician, and the map user’s intended beneficiary. Only the Likert-scale satisfaction question was mandatory, meaning that respondents were not required to answer any other questions to submit their survey responses. The survey was developed using an iterative process whereby question sets were tested internally by the research team and refined accordingly. Study data were collected and managed using the Research Electronic Data Capture (REDCap) electronic data capture tools hosted at the Institut du Savoir Montfort [10,11]. REDCap is a secure, web-based software platform designed to support data capture for research studies, providing (1) an intuitive interface for validated data capture, (2) audit trails for tracking data manipulation and export procedures, (3) automated export procedures for seamless data downloads to common statistical packages, and (4) procedures for data integration and interoperability with external sources. The survey was pilot-tested by the research team and external colleagues. Because the survey consisted of only 2 questions, validity and reliability were not evaluated.

Data about total website visits to the tool were also collected using Google Analytics during the study period. Google Analytics collects and reports only anonymized, aggregated, and unidentifiable observations about users’ interactions with public websites [12] and has previously been used in studies of online resources for mental health [13], nursing [14], cardiology [15], sexual health [16], and dermatology [17].

### Ethical Considerations

This study was approved by Hôpital Montfort Research Ethics Board (application number 22-23-09-033). Participants provided informed consent before completing the survey and responses were anonymous. Participants were not compensated for participating.

### Analyses

Likert-scale responses are reported as summary statistics. We evaluated responses to the question “On a scale of one (1) to five (5), how would you rate your overall appreciation of the map?” by first collapsing responses into three categories: “very satisfied/somewhat satisfied,” “neither satisfied nor dissatisfied,” and “very dissatisfied/somewhat dissatisfied.” We then evaluated responses for the population as a whole and compared responses between demographic groups using the Pearson χ^2^ test with the chisq.test() function in the R Language for Statistical Computing [18].

We analyzed short-answer responses to the question “Please give us any suggestions for improvement that you might have regarding the map” using thematic analysis. The coding scheme was initially developed by one author (CB) inductively from a close reading of the responses, and the final scheme was revised and finalized through discussion and consensus with two other authors (CP and LMB). The three authors (CB, CP, and LMB) coded each response individually and then met to discuss and arrive at a consensus coding for each response. Responses were assigned more than one code if multiple themes were present.

## Results

### Demographics

There were a total of 3726 unique “new user” visits to the English and French versions of the online map during the study period. User demographics are presented in Table 1. Approximately 91% of all visits were from users in Ontario or Quebec (ie, in or near the study’s intended population base).

**Table 1:** New user demographics from Google Analytics November 9, 2022, to June 19, 2023 (N=3726).

Characteristics			Users, n (%)
**Country**			
	Canada	3456 (92.8)	
	United States	186 (5.0)	
	China	16 (0.4)	
	United Kingdom	12 (0.3)	
	France	10 (0.3)	
	Other	46 (1.2)	
**Region**			
	Ontario	2598 (69.7)	
	Quebec	795 (21.3)	
	(not set)	65 (1.7)	
	Virginia	64 (1.7)	
	British Columbia	18 (0.5)	
	Other	186 (5.0)	
**Language**			
	English	3205 (86.0)	
	French	483 (13.0)	
	Other	12 (1.0)	

The survey received 103 responses, of which 102 were submitted and marked “complete” in REDCap. Of the 102 complete responses, 92 included a valid postal code in Ontario or Quebec, and one respondent who did not provide a valid postal code self-identified as living in the Ottawa region in their free-text response. These 93 respondents constitute our final sample for a response rate of approximately 2.7% of Ontario- and Quebec-based site visitors. Respondent demographics are shown in Table 2.

**Table 2:** Demographics for valid completed surveys (N=93).

Characteristic		Respondents, n (%)
**Age group (years)**		
	<18	1 (1.1)
	18 to 29	11 (11.8)
	30 to 44	17 (18.3)
	45 to 54	13 (14.0
	55 to 64	28 (30.1)
	65 to 74	18 (19.4)
	≥75	2 (2.2)
	Not specified	3 (3.2)
**Preferred language**		
	English	54 (58.1)
	French	36 (38.7)
	Other	1 (1.1)
	Not specified	2 (2.2)
**Has a family physician**		
	Yes	33 (35.5)
	No	57 (61.3)
	Not specified	3 (3.2)

### Overall Satisfaction

Overall, respondents reported that they were satisfied with the tool. Among the 93 respondents, 57 (61%) reported they were “very satisfied” or “somewhat satisfied,” 16 (17%) reported they were “neither satisfied nor dissatisfied,” and 20 (22%) reported they were “very dissatisfied” or “somewhat dissatisfied.” We found no statistically significant differences when comparing responses between groups based on preferred language (*P*=.24*)*, age group (*P*=.57), whether they had a family physician (*P*=.09), and who they were using the map for (*P*=.66) using the Pearson χ^2^ test.

### Qualitative Feedback

#### Themes Overview

Our survey received 56 short-answer responses to the open-ended question “Please give us any suggestions for improvement that you might have regarding the map” (n=44 English responses, n=12 French responses). Responses were categorized using six codes grouped into three themes: *General Feedback* on the map, comments about the *Physician Information* included in the map, and comments about participants’ difficulties *Finding a Family Physician.* Our full data structure is provided in Table 3, and each theme and code are described in more detail below. Table 3 lists the major themes and illustrates them with representative quotations.

**Table 3:** Thematic analysis data structure.

Theme and code			Description		Representative quote^a^		Respondents
**General feedback**							
	Positive feedback	Positive comments about the map with no specific suggestions		“Facile a voir les coordonnées et endroits” (*Easy to see coordinates and locations*) “Excellente initiative! Super travail!” (*Excellent initiative! Great work!*)		5	
	User interface improvement	Suggestions for improving the user interface, including the interface with the survey		“ask for comments after more than one ‘zoom in’” “Once you read the instructions they are no longer needed so should be able to minimize or close”		23	
**Physician information**							
	Currency of data	Comments related to keeping the physician database up to date		“Keep it up to date” “The information is as out of date as the doctors website.”		6	
	Additional languages	Suggestions to include additional languages in the selection menu		“You should include Indigenous languages, at least for First Nations and Inuit.”		2	
	Accepting new patients	Comments expressing a desire to know which physician offices are accepting new patients		“The whole point of accessing this map was to find doctors accepting new patients, which the map doesn’t show!” “The map is good but it doesn't give any information about available family doctors who accepts new patients. So it is not that useful”		20	
Inability to find a family physician			Expressions of participants’ inability to find a family physician		“nothing works and I cannot find a doctor. I have been without a family doctor since 2019... I'm very nervous” “Formore than one year I am looking for a family Dr and I CANNOT find one at all.”		5

^a^Quotes are reproduced verbatim as written by respondents and may include abbreviations or typos. French quotations are followed by the authors’ English translations in parentheses.

#### General Feedback

The most common type of general feedback (n=23) we received was suggestions for *user interface improvements.* Some users reported experiencing technical issues with the user interface, including that the map kept “freezing.” Some respondents experienced difficulties using the map on smaller screens, reporting that it was “not iPhone friendly” and “hard to pinpoint the locations on mobile phone.” Other users suggested improvements to the site layout and to the survey itself, suggesting, for example, that the survey should be provided “AFTER someone has had a chance to view the map.” A smaller number of respondents (n=5) provided *positive feedback* on the map, noting, for example, that it was “Facile a voir les coordonnées et endroits” (Easy to see coordinates and locations).

#### Physician Information

Many users commented on the physician information that was and was not contained within the map. The most common issue was respondents’ dissatisfaction that the map did not contain information about which physicians were accepting new patients (n=20). For some respondents, the map itself was of questionable utility without this information: “the only reason one would want to use this tool is to see which doctors are taking new patients, so that fact that you don't filter by this makes this information utterly irrelevant.” In some cases, not knowing which physicians were accepting new patients could create negative experiences: “Knowing that there are 5 physicians speaking my language BUT none of them are available seems to only add to the frustration.”

Respondents also commented on the timeliness and completeness of the data contained within the map. Some noted *data currency* (n=6) as an issue, suggesting that the data were “out of date - seriously.” A small number (n=2) suggested including *additional languages* in the map, including Indigenous languages.

#### Finding a Family Physician

The last theme we identified was *Finding a Family Physician,* in which several (n=5) respondents shared personal stories of their difficulties in finding family physicians. Some participants shared straightforward statements of need, such as “I need a family doctor.” However, others shared emotional stories of living with medical complexity and the difficulty of finding language-concordant health care when a long-time physician retires: “Notre médecin a pris sa retraite et sa remplaçante est unilingue anglais et est incapable de comprendre les rapports médicaux en français!!!” (Our doctor retired and his replacement only speaks English and is incapable of understanding medical reports in French!!!).

## Discussion

### Summary of the Findings

We found that most users were satisfied with the mapping tool, suggesting that this kind of online, interactive, map-based data source is valuable for many patients. At this formative stage, we believe that these results are sufficient to show that our tool has real value for some patients. Furthermore, many respondents who were not satisfied expressed dissatisfaction that the tool did not contain information about which family physicians were accepting new patients. In fact, our qualitative findings suggest that many patients’ primary interest in the map was to help them find a local language-concordant family physician who was also accepting new patients. This suggests that the map would be more satisfactory to a significant subset of patients if it included this information.

### Explanation of the Findings

Our findings suggest that there could be significant patient value in reporting which Ontario physicians are accepting new patients. However, at present, there is no centralized source for these data in the region; in other jurisdictions such as Norway, this has become the main means of finding and securing a family physician [19]. There are currently no reporting requirements for family physicians to disclose this information nor is it reported in the College of Physicians and Surgeons of Ontario’s physician information directory or, to our knowledge, by the Ontario Ministry of Health [20]. As such, there is no ready-at-hand data source that could be incorporated into the mapping tool.

Although our focus was on evaluating the mapping tool, our qualitative findings also align with the research literature on the importance of access to language-concordant health care [1-3], further underscoring the need for better access to and information about these services. Some participants also expressed a desire to have more languages featured in the map; however, since the map shows all languages spoken by family physicians within the study region, this limitation reflects the language competencies reported by physicians.

Our quantitative analysis found no statistically significant differences between demographic groups, for which we suggest two potential explanations. First, our study’s relatively small sample size may mean that it was underpowered to detect any significant differences between groups. Second, there was relative homogeneity in users’ overall experiences using the mapping tool.

### Future Directions

Future research could explore the topic of which physicians are accepting new patients in several ways. First, additional patient-focused studies could explore patients’ perceptions and needs for this information to validate its general utility. Second, physician-focused studies could explore physician and office staff perspectives on making this information available. Applied research could incorporate both patients’ and physicians’ perspectives to explore ways of collecting and disseminating this information. Finally, more theoretical and policy-oriented research could examine policy options to bridge these identified gaps in the context of Ontario’s health care system.

### Limitations

This study has several limitations. First, although we have taken steps to ensure all respondents were within the intended study population and there was no indication of malicious or false responses, we used an anonymous online survey, which could be subject to manipulation. Second, our sample is limited to those who visited the website and elected to fill out the survey; therefore, our sample may be biased toward users with greater computer literacy and health literacy. Finally, our sample size of 93 represents a response rate of 2.7% of the 3393 website visitors from Ontario and Quebec, meaning that care must be taken in applying our findings to the full population.

### Conclusion

We conducted a cross-sectional online survey of people who accessed an online interactive map of family physicians in Ontario. While most patients were satisfied with the online map, a significant minority expressed dissatisfaction that the map did not show which family physicians were accepting new patients. This suggests that there may be public interest in an accessible database of which family physicians in Canada are currently accepting new patients.

## Appendix

Multimedia Appendix 1 Study pop-up advertisement.

Multimedia Appendix 2 Survey protocol used in the study.

## Abbreviations

REDCap: Research Electronic Data Capture

## Data Availability

Data from this study cannot be shared at this time for ethical reasons.
