# Supplementary material for: Patient and Provider Satisfaction With a Geomapping Tool for Finding Community Family Physicians in Ontario, Canada: Cross-Sectional Online Survey Study
Source: JMIR Form Res. 2024 Jul 9;8:e56716. doi: 10.2196/56716 (PMC11267088; doi:10.2196/56716)
Supplement: Multimedia Appendix 1 [file formative_v8i1e56716_app1.docx]

## Multimedia Appendix 1: Study Pop-up Window Advertisements

English Pop-Up Window Advertisement

**
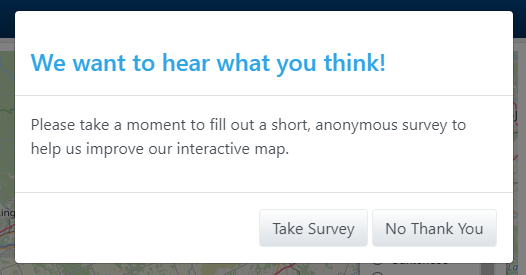
**

French Pop-Up Window Advertisement

**
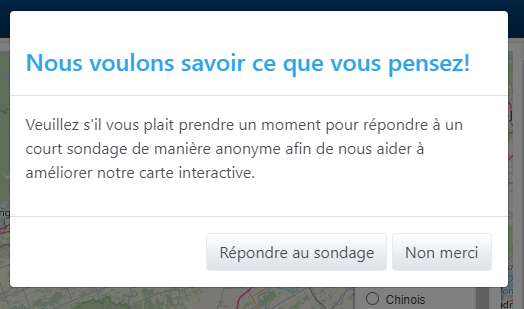
**
