# Supplementary material for: Patient and Provider Satisfaction With a Geomapping Tool for Finding Community Family Physicians in Ontario, Canada: Cross-Sectional Online Survey Study
Source: JMIR Form Res. 2024 Jul 9;8:e56716. doi: 10.2196/56716 (PMC11267088; doi:10.2196/56716)
Supplement: Multimedia Appendix 2 [file formative_v8i1e56716_app2.docx]

## Multimedia Appendix 2: Survey Protocol

Section A: Satisfaction with the map

1. On a scale of zero (0) to five (5), how would you rate your overall appreciation of the map?

1: Very dissatisfied

2: Somewhat dissatisfied

3: Neither satisfied nor dissatisfied

4: Somewhat satisfied

5: Very satisfied

2. Please give us any suggestions for improvement that you might have regarding the map. *(free text response)*

Section B: Respondent characteristics

Prompt text: To help us in making the map better for our users, we would appreciate it if you could tell us a bit about your characteristics. NOTE: None of this information can be used to identify you as a person, and all information gathered will be handled confidentially. If you do not wish to answer a question, simply press “NEXT” and skip to the next question.

3. Which age group do you belong to?

- 18-29

- 30-44

- 45-54

- 55-64

- 75 years or older

4. What is your preferred language?

- English

- French

- Other (free text box)

Section C: Patient demographics

5. What are the first three digits of your postal code? (Free text box)

6. Who are you using the map for?

- yourself

- a friend

- a family member

- a patient (if you are a health professional)

Section D: Health status and access to care

To help us better understand the needs of our map users, please tell us about your state of health and your access to the healthcare system.

7. Are you currently attached to/registered with a family doctor or nurse practitioner?

-yes

- no

8. How many times have you visited a family physician in the past year? (including in a walk-in clinic)

- 0

- 1-2

- 3-4

- 5-6

- 6+

9. How many prescription medications do you currently take?

- 0

- 1-2

- 3-4

- 5-6

- 6+
